# Exploration of the skeletal phenotype of the *Col1a1* ^+/Mov13^ mouse model for haploinsufficient osteogenesis imperfecta type 1

**DOI:** 10.3389/fendo.2023.1145125

**Published:** 2023-03-08

**Authors:** Lauria Claeys, Lidiia Zhytnik, Lisanne E. Wisse, Huib W. van Essen, E. Marelise W. Eekhoff, Gerard Pals, Nathalie Bravenboer, Dimitra Micha

**Affiliations:** ^1^ Department of Human Genetics, Amsterdam Movement Sciences, Tissue Function & Regeneration and Musculoskeletal Health, Amsterdam University Medical Centers (UMC) location Vrije Universiteit Amsterdam, Amsterdam, Netherlands; ^2^ Department of Traumatology and Orthopeadics, Institute of Clinical Medicine, The University of Tartu, Tartu, Estonia; ^3^ Department of Clinical Chemistry, Amsterdam Movement Sciences, Tissue Function & Regeneration and Ageing & Vitality, Amsterdam University Medical Centers (UMC) location Vrije Universiteit Amsterdam, Amsterdam, Netherlands; ^4^ Department of Endocrinology and Metabolism, Amsterdam Rare Bone Disease Center, Amsterdam University Medical Centers (UMC), Amsterdam, Netherlands

**Keywords:** Mov13, osteogenesis imperfecta, *Col1a1*, haploinsufficient, bone, collagen, mouse model, skeletal dysplasia

## Abstract

**Introduction:**

Osteogenesis Imperfecta is a rare genetic connective tissue disorder, characterized by skeletal dysplasia and fragile bones. Currently only two mouse models have been reported for haploinsufficient (HI) mild Osteogenesis Imperfecta (OI); the *Col1a1* ^+/Mov13^ (Mov13) and the *Col1a1* ^+/-365^ mouse model. The Mov13 mice were created by random insertion of the Mouse Moloney leukemia virus in the first intron of the *Col1a1* gene, preventing the initiation of transcription. Since the development of the Mov13 mice almost four decades ago and its basic phenotypic characterization in the 90s, there have not been many further studies. We aimed to extensively characterize the Mov13 mouse model in order to critically evaluate its possible use for preclinical studies of HI OI.

**Methods:**

Bone tissue from ten heterozygous Mov13 and ten wild-type littermates (WT) C57BL/6J mice (50% males per group) was analyzed at eight weeks of age with bone histomorphometry, micro computed tomography (microCT), 3-point bending, gene expression of different collagens, as well as serum markers of bone turnover

**Results:**

The Mov13 mouse presented a lower bone strength and impaired material properties based on our results of 3-point bending and microCT analysis respectively. In contrast, no significant differences were found for all histomorphometric parameters. In addition, no significant differences in *Col1a1* bone expression were present, but there was a significant lower P1NP concentration, a bone formation marker, measured in serum. Furthermore, bone tissue of Mov13 mice presented significantly higher expression of collagens (*Col1a2*, *Col5a1* and *Col5a2*), and bone metabolism markers (*Bglap*, *Fgf23*, *Smad7*, *Edn1* and *Eln*) compared to WT. Finally, we measured a significantly lower *Col1a1* expression in heart and skin tissue and also determined a higher expression of other collagens in the heart tissue.

**Conclusion:**

Although we did not detect a significant reduction in *Col1a1* expression in the bone tissue, a change in bone structure and reduction in bone strength was noted. Regrettably, the variability of the bone phenotype and the appearance of severe lymphoma in adult Mov13 mice, does not favor their use for the testing of new long-term drug studies. As such, a new HI OI type 1 mouse model is urgently needed.

## 1 Introduction

Osteogenesis Imperfecta (OI), also known as brittle bone disease, is a rare connective tissue disorder affecting 1 in 15-20,000 births (1). It is mainly characterized by skeletal fragility and dysplasia, substantial growth deficiency, blue sclerae and early-onset hearing loss. OI can present multiple genetic causes, most of which are directly related to collagen type I synthesis and regulation. Approximately 85 to 90% of the patients have autosomal dominant OI caused by pathogenic variants α1(I) collagen gene (*COL1A1)* and α1(II) collagen gene (*COL1A2)* (2). These genes code for the α1(I) and α2(I) chains of collagen type I, which is the most abundant protein of bone, skin and tendon extracellular matrices. Other genetic causes include pathogenic variants in genes coding for proteins that facilitate collagen folding or post-translational modification and other aspects of bone metabolism (3).

Collagen type I defects can be divided into two groups, one showing less collagen type I production (haploinsufficiency (HI)) and the other showing abnormal collagen type I structure (dominant negative (DN)). HI mainly causes mild OI whereas the DN defects mostly cause severe OI forms, which highlights the necessity for distinct mouse models for their study. At the moment, multiple mouse models are in existence for OI, but only two are available for HI in mild OI (Sillence type 1), which represents the majority of the patients (4–6). The Mov13 model was developed by Jaenish et al. in 1983 and characterized by Bonadio et al. and Jepsen et al. (7–13) It was created by microinjection of the Moloney murine leukemia virus (MMuLV) at midgestation of C57BL/6 mice (7). The virus integrated in the first intron, 19 bp away from the splice donor site of the *Col1a1* (9). The insertion prevents initiation of *Col1a1* transcription by causing a change in the chromatin conformation and *de novo* methylation of the gene (12). In the case of homozygous embryos this leads to a lethal developmental arrest between day 11 and 12 of gestation, while heterozygous embryos serve as a model for mild HI OI (9, 10). Heterozygous Mov13 mice are reported to show around 50% reduction in type I collagen in non-mineralized connective tissue and display reduced mechanical and material properties in the long bones (10, 14). In addition, the mice also present progressive hearing loss, resembling the human phenotype (10, 11).

As the bone phenotype is the most pronounced feature in OI, Mov13 mouse studies have been mostly focused around long bone mechanical and histological properties (10, 11, 15). The brittleness of the bones has been explained by altered microstructure, reduced ductility and increase in porosity (11). Studies in skin, revealed approximately 50% reduction in collagen type I and thin collagen fibers (10, 16). Aside from the bone and skin, limited research has been performed on other collagen type I-rich tissues presenting the OI phenotype. Otopathology analyses determined hearing loss which appears to be connected with the cochlear hair cell loss in relation to bony changes (10, 17). In addition, Stankovic et al. confirmed a 50% reduction in *Col1a1* expression in the otic capsule (18). Derwin et al. studied the mouse tail tendon fascicle, which revealed reduced fibril size but no change in tissue mechanics (19, 20). A histological study of homozygous Mov13 embryos revealed that the rupture of major blood vessels between day 12 and 14 during the gestational was the cause of their lethality, due to the lack of collagen type I in combination with increased blood pressure at that age (21). Though observed in patients with OI type 1, dentinogenesis imperfecta and impaired stability of the aorta and the cardiac valves have not been well characterized in Mov13 mice. Concerning dentinogenesis, tooth germ cells taken from homozygous Mov13 embryos were capable of differentiating into all dental tissues and odontoblasts produced a normal layer of dentin (22). Moreover, Kratochwil et al. failed to identify a transcriptional block in the odontoblasts of Mov13 mice which were determined to produce normal amount of collagen type I (22).

It has been estimated that Sillence type 1 OI represents ~ 46–71% of the whole OI population; in approximately 40% of type 1 patients OI is caused by *COL1A1* HI (23, 24). Even though the disease in HI patients is milder regarding skeletal deformities, these patients can still experience a vast number of fractures. A cohort of 86 young HI OI patients showed a 95-fold increase in long-bone fracture risk (24). Another study of 364 Italian OI patients showed that regarding the HI OI type 1 patients ~48% showed osteopenia and ~38% had osteoporosis. Also, 28% presented deafness and in 24% cardiac defects were recorded (25). Thus, considering the high number of these patients, the impact of OI on their quality of life and lack of therapy, the existence of a reliable mouse model is paramount to adequately study the underlying pathology of HI OI. Based on the above, it is evident that phenotyping studies of Mov13 mice are mostly limited to bone and skin tissue. Replicability of these results and further investigation of the phenotype is required with extended methodological approaches to be able to use this model in future studies on new therapies for HI OI type 1. In an effort to address this need, we performed an in-depth characterization of the bone phenotype of Mov13 mice regarding their histomorphological and gene expression properties in order to provide a reference for the suitability of this model for testing of new therapies and long-term drug studies.

## 2 Materials and methods

### 2.1 Animal & ethics claim

Male and female mice, heterozygous for the insertion of MMuLV in the *Col1a1* locus, were purchased from Jackson Laboratories (USA) and mated to obtain ten heterozygous Mov13 mice (50% males) and ten wild-type (WT) (C57BL/6J) littermates (50% males). All animal experiments were approved by the Central Committee of Animal experiments (CCD) of the Netherlands and in agreement with the Animal Welfare Body of the Amsterdam UMC in full compliance with the directive 2010/63/EU.

### 2.2 Tissue preparation for histochemistry, microCT and 3-point bending

Four-week-old mice were weighted twice a week for four weeks and sacrificed at eight weeks of age by CO_2_-induced hypoxia. Blood serum, humeri, femora, tibiae, the ventricles of the heart and abdominal skin tissue were obtained immediately after.

Blood was collected by heart puncture. Serum was acquired by centrifuging the blood at 4°C twice for 10min at 1000g and stored at -20°C. Both humeri and the right femur were centrifuged, after being cleaned of soft tissue, to remove bone marrow, before storing in RNAlater™-ICE solution (Thermo Fisher Scientific, Waltham, MA, US) at -20°C for gene expression measurements. Immediately after extraction, both heart and skin tissue were also stored in RNAlater™-ICE solution at -20°C.

The cleaned left femora were stored at 4°C in a PBS-soaked gauze before micro computed tomography (microCT) analysis and consecutive 3-point bending test. The cleaned, undecalcified left tibiae were stored in 70% ethanol and processed for methylmethacrylate (MMA) embedding. MMA (BDH Chemicals, Poole, UK) was supplemented with 20% dibutyl phtalate (Merck, Darmstadt, Germany), 8.0 g/l dibenzoyl peroxide (AKZO Nobel, Deventer, The Netherlands), and 22 ml/10 ml N,N-dimethyl-p-toluidine (Merck). Sections of 5µm thickness were cut with a Leica Polycut SM 2500S microtome (Leica Microsystems, Wetzlar, Germany) and mounted onto gelatin-coated glass slides (VWR, Super premium microscope Slides).

### 2.3 Histomorphometry analysis

Left tibia MMA-embedded sections were stained following standard protocols for Masson-Goldner trichrome, Von Kossa and tartrate-resistant acid phosphatase (TRAP) staining for analysis of bone microarchitecture and turnover, and Safranin O staining for analysis of cartilage. Bright field images were captured using a microscope Nikon eclipse E800 (Nikon Instruments Inc., Melville, NY, US) and camera Nikon digital sight DS-5Mc (Nikon Instruments, Inc., Melville, NY, US) for Safranin O staining and Olympus SLIDEVIEWTM VS200 Slide Scanner (Olympus Corp., Tokyo, Japan) for Masson-Goldner trichrome, Von Kossa and TRAP staining. Images were analyzed using the NIS-Elements Imaging Software, version AR 4.10.01 (Nikon Instruments Inc., Melville, NY, US).

Sections stained for Safranin O (n=2 sections per mouse (eight WT and ten Mov13)) were measured for the length, thickness, column index and percentage of cells in columns. Images were captured at 10x10 magnification. The length of the growth plate was measured by following the middle of the growth plate (light green line on **Supplemental Figure 1**). The thickness was measured in five different places perpendicular to the top of the growth plate. The three different zones (resting zone (RZ, dark green, **Supplemental Figure 1**), proliferative zone (PZ, dark blue, **Supplemental Figure 1**) and hypertrophic zone (HZ, light blue, **Supplemental Figure 1**) were similarly measured at the same location within the growth plate. The ratio of the different regions was presented as a percentage of the total thickness of the growth plate. A column was defined as: 1) Being composed of a minimum of 3 cells, 2) the angle between each cell in the column must range from -155 to -179° or 155 to 180° (blue and green arrow on **Supplemental Figure 1**) and 3) the cells cannot be more than 20 pixels apart. The length of the column was measured by using the distance between each cell starting from the center of the cell (yellow line on **Supplemental Figure 1**). The percentage of cells in columns was counted manually by dividing the number of cells counted in the columns (green triangles on **Supplemental Figure 1**) by the total amount of cells counted (cells in column + cells outside column (yellow dots) in **Supplemental Figure 1**). Afterwards the column index was calculated by multiplying the percentage of cells in columns with the average column length measured (26).

Masson-Goldner trichrome, TRAP and Von Kossa analyses were performed (n=2 sections per mouse (ten WT and ten Mov13 mice)). For Goldner trichrome and TRAP-stained slides 10x objective magnification was used, and for Von Kossa-stained slides 20x objective magnification was used. Primary measured parameters for Goldner trichrome included cortical width (Ct.Wi), tissue area (T.Ar), bone area (B.Ar), bone perimeter (B.Pm), osteocyte number (N.Ot); osteoclast number (N.Oc), osteoclast perimeter (Oc.Pm) for TRAP; for Von Kossa this included osteoid perimeter (O.Pm) and osteoid width (O.Wi). Cortical width (Ct.Wi) were measured four times for both sites of each section following a spacing grid of 200µm, at least 200-400µm distance from the growth plate. The trabecular bone was assessed in a defined area between 200-400µm from the growth plate, and half of Ct.Wi from the cortical bone, in order to exclude primary spongiosa and endocortical bone. All measurements and analysis were performed according to the American Society for Bone and Mineral Research (ASBMR) nomenclature committee (27).

### 2.4 Micro computed tomography

The left femora were scanned using the Scanco Medical AG microCT scanner (MicroCT42, version 6.1). Scans were automatically reconstructed in 2D slices and were analyzed using the Skyscan CT-analyzer (version 6.6). A trabecular section of 2.37mm (395 slices) of the distal left femur and a cortical section of 1.11mm (185 slices) of the midshaft were scanned. Femora were placed vertically in a 11.5mm scanner holder and scanned with the following settings: 10µm voxel size, 55kV_p_ and 145μA, with an integration time of 200ms. Three-dimensional reconstruction and analysis were performed with CTan software (Bruker, Billerica, MA, USA, version 1.13.11.0). The trabecular bone parameters measured include trabecular bone volume fraction (Tb. BV/TV), trabecular bone surface density (Tb.BS/TV), trabecular thickness (Tb.Th), trabecular number (Tb.N), trabecular separation (Tb.S) and connectivity density (Conn.D). For cortical bone, the total cross-sectional area inside the periosteal envelope (Tt.Ar), cortical bone area (Ct.Ar), cortical area fraction (Ct.Ar/Tt.Ar), average cortical thickness (Ct.Th), cortical porosity (Ct.Po), total pore volume (Po.V) and pore density (Po.Dn) were measured. The cortical thickness was measured as the average measurement between the outer edge of the periosteum and the endosteum with the pores filled in. Both the trabecular and cortical parameters were corrected for body weight using the linear regression method as described in Jepsen et al. (28)

### 2.5 Mechanical testing

After microCT the mechanical properties of the left femora were measured by three-point bending analysis with the Instron E1000 (Electrodynamic Testing System V1.4 Upgrade, United Kingdom). Each bone was placed on two supports, that have a span of 7mm, with the anterior surface facing down and a transverse force was applied at the mid-diaphysis at 5mm/s until failure occurred. Force (F) and displacement (d) data were collected with the WaveMatrix 2 (Instron, version 2.0) software and used for the calculation of the ultimate load (N), breaking load (N), stiffness (N/mm), yield load (N), post-yield displacement (mm) and work-to-fracture (N/mm). These mechanical properties were calculated using standard definition and corrected for midshaft total area (Tt.Ar) measured with microCT using the linear regression method as mentioned before in Jepsen et al. (**Supplemental Figure 2**) (28).

### 2.6 ELISA

Blood was collected from eight-week-old heterozygous Mov13 (n=10) and WT littermates (n=10). After isolation, the serum was analyzed for the levels of Bone Turnover Markers (BTM), procollagen type I N-terminal propeptide (P1NP) (P1NP EIA kit, immunodiagnostic systems, AC-33F1) and Tartrate-resistant acid phosphatase 5b (TRAcP 5b) (TRAcP 5b, immunodiagnostic systems, SB-TR103), by enzyme-linked immunosorbent assay (ELISA) according to manufacturer’s protocol.

### 2.7 Quantitative real-time PCR

Humeri and right femora of nine Mov13 (60% males) and ten WT littermates (50% males) C57BL/6J mice were combined and pulverized in liquid nitrogen with the 6775 Freezer/Mill^®^ Cryogenic Grinder (SPEX Sample Prep, Metuchen, NJ, US). Total bone RNA was extracted first with TRIzol^®^ Reagent (Thermo Fisher Scientific, Waltham, MA, US) and then isolated using a RNeasy kit (Qiagen, Hilden, Germany). Heart ventricle RNA and abdominal total skin RNA (ten Mov13 and ten WT) was isolated using the RNeasy kit (Qiagen, Hilden, Germany) according to the manufacturer’s protocol. RNA concentrations were measured with Nanodrop spectrophotometer (NanoDrop Technologies, Wilmington, NC, US) and cDNA was synthesized from 140 ng of RNA with a SuperScript VILO kit (Thermo Fisher Scientific, Waltham, MA, US) in 20 µl final volume. The statistical expression of *Col1a1, Col1a2*, other collagens and tissue-specific markers was tested by quantitative real-time PCR (qPCR). qPCR analysis was performed in 10µl volume in duplicates with LightCycler^®^ 480 SYBR Green I Master (Roche, Basel, Switzerland) and performed with the LightCycler^®^ 480 System (Roche, Basel, Switzerland) according to the following program: 10 min at 95°C; 45 cycles consisting of 10s at 95°C, 5s at 60°C, 10s at 72°C, 5s at 78°C; 1 cycle of 1s at 95°C, 1s at 60°C and 1s at 95°C; and 30s at 40°C. List of used primers is presented in **Table 1**. Raw data was analyzed with the LightCycler^®^ 480 Software, version 1.5 (Roche, Basel, Switzerland). Expressions of target genes were normalized towards the housekeeping TATA-box binding protein (*Tbp*) gene The relative gene expression was calculated with the ΔΔCt method.

**Table 1:** Primers used in qPCR analysis and tissue, in which corresponding gene expression was tested.

Gene	Forward	Reverse	Product length (bp)	Tissue tested
*Col1a1*	CGATGGATTCCCGTTCGAGT	TTCGATGACTGTCTTGCCCC	292	Bone, heart, skin
*Argrt1a*	TAGGGTTGGAACCTGCGGA	TGTTCAAAATGCACTTGATCTGG	321	Heart
*Bglap*	TTCTGCTCACTCTGCTGACC	GGGACTGAGGCTCCAAGGTA	154	Bone
*Cd105*	TTGCACTTGGCCTACGACTC	ATGCTTTGGGGGTCATCCAG	161	Bone
*Cd11b*	GCTCGACACCATCGCATCTA	CCCAGCAAGGGACCATTAGAG	204	Bone
*Cd45*	TCCTCGTCCACTGCAGAGAT	GTCCATTCTGGGCGGGATAG	210	Bone
*Cgref1*	AAGGATGGAGTTGCAAGGTTGG	CCGGGTTGATGGGAAAGTGT	277	Bone, heart
*Col10a1*	GCATCTCCCAGCACCAGAATC	GTGTCTTGGGGCTAGCAAGT	153	Bone, heart, skin
*Col1a2*	AATGGTGGCAGCCAGTTTGA	TCCAGGTACGCAATGCTGTT	143	Bone, heart, skin
*Col3a1*	GAGGAATGGGTGGCTATCCG	GCGTCCATCAAAGCCTCTGT	314	Bone, heart, skin
*Col5a1*	TGGGGAGAGCTACGTGGATT	CAAGAAGTGATTCTGGCTCCC	294	Bone, heart, skin
*Col5a2*	AGCAGTTGGCCCATTAGGAC	TCTCTCCTATGCCACCTCGG	248	Bone, heart, skin
*Tnnt2*	CAACATGATGCACTTTGGAGGG	TCCCACGAGTTTTGGAGACTTT	293	Heart
*Cyp2e1*	GTCATCCCCAAGGGTACAGT	AGGCCTTCTCCAACACACAC	182	Bone, heart
*Edn1*	CGTCGTACCGTATGGACTGG	GATGGCCTCCAACCTTCGTA	283	Bone, heart
*Eln*	TGGAGCTGCTGGAGGTTTAGT	CTCCACCTCTGGCTCCGTATT	218	Bone, heart
*Fbn1*	GGGCAGAGACTGTGGGTG	TAGTCCCTGCCTGTCTGGA	108	Bone
*Fgf23*	CCCATCAGACCATCTACAGTGC	TCGCGAGAGCAGGATACAGG	433	Bone
*Fn1*	GAGTGGAAGTGTGAGCGACA	CGAGTCTGAACCAAAACCGC	431	Bone
*Gapdh*	GTGCTGAGTATGTCGTGGAG	TCGTGGTTCACACCCATCAC	142	Housekeeping
*Gata4*	CAATGCGGAAGGAGGGGATT	ACACAGTACTGAATGTCTGGGA	243	Heart
*Gypa*	CCCAGTATGACCGAGAGCAC	TCAGTAGGGGCCGTGTGATA	242	Heart
*Ifitm5*	CCCTCTCCATGGGAACACCT	TCTTCTGGTCTCGGGCCTTG	135	Heart
*Il10*	GGCCCAGAAATCAAGGAGCA	AGACACCTTGGTCTTGGAGCTTAT	160	Bone, heart
*Il1b*	TGCCACCTTTTGACAGTGATG	AAGGTCCACGGGAAAGACAC	220	Bone, heart
*Il6*	GCCTTCTTGGGACTGATGCT	TGCCATTGCACAACTCTTTTC	181	Bone, heart
*Spp1*	CTGGCTGAATTCTGAGGGACT	TTCTGTGGCGCAAGGAGATT	207	Bone, heart
*Tnfsf11* *(Rankl)*	TACTTTCGAGCGCAGATGGA	CCAGAGTCGAGTCCTGCAAA	109	Bone
*Rerg*	ATGGAGATCCTGGACACCGC	CCTCGATCGGTGATGTCGTA	104	Heart
*Runx2*	GCGGTGCAAACTTTCTCCAG	ACTGCTTGCAGCCTTAAATATTCC	149	Bone
*Serpine1*	ATCGAGGTAAACGAGAGCGG	GAAGAGGATTGTCTCTGTCGGG	141	Bone, heart
*Slc13a5*	TTTGCCTCCATGGCTCGTT	AATTTGCCCAGTCGGGGAAG	262	Heart
*Smad7*	TTTTCTCAAACCAACTGCAGGC	AATTGAGCTGTCCGAGGCAA	257	Bone, heart
*Smpd3*	CTCTGTTTCTCAAGGTGCAGGT	AGCGAGTAAAGAGCGAGTGC	285	Heart
*Sost*	ATCTGCCTACTTGTGCACGC	GTACTCGGACACATCTTTGGC	201	Bone
*Tbp*	CCTATCACTCCTGCCACACC	ATGACTGCAGCAAATCGCTTG	161	Housekeeping
*Tbx20*	ACCATCAAACCCCTGGAACAA	GTGGTGGGTATCAGTGGCTC	152	Heart
*Tgfβ1*	ACTGGAGTTGTACGGCAGTG	GGGGCTGATCCCGTTGATT	123	Bone, heart
*Tnfα*	AGGCACTCCCCCAAAAGATG	CCATTTGGGAACTTCTCATCCC	157	Bone, heart
*Tnfrsf11b* *(Opg)*	CGCACTCCTGGTGCTCCT	ACACTGGGCTGCAATACACA	239	Bone

### 2.8 Statistics

All data were tested for a normal distribution with the Kolmogorov-Smirnov test and analyzed using the unpaired T-test in GraphPad Prism version 9 (GraphPad Software Inc., San Diego, CA, US). For analysis of the body weight on the different timepoints a two-way ANOVA corrected for time and with multiple comparisons was used. A P-value of ≤ 0.05 indicated the statistically significant difference. Graphs, including Heatmaps were generated using GraphPad Prism version 9 (GraphPad Software Inc., San Diego, CA, US). For Heatmaps values of ΔΔCt were used.

## 3 Results

### 3.1 Reduced body weight in male Mov13 mice

**Figure 1:**
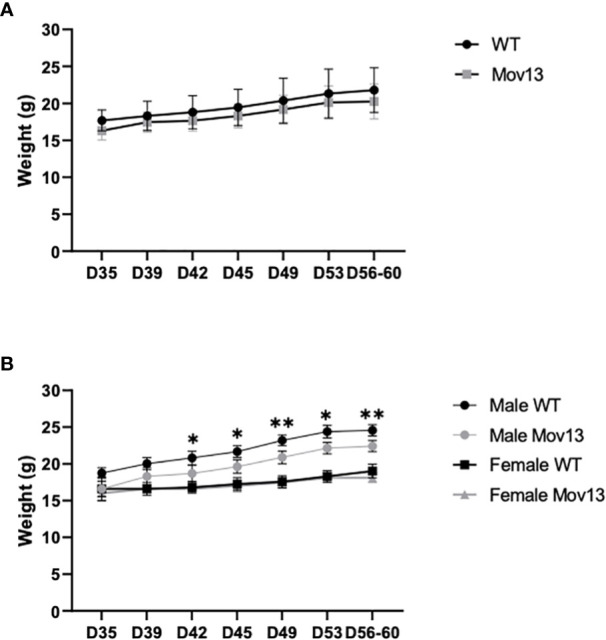
Weight of four-week-old till eight-week-old mice. **(A)** The weight of four-week-old Mov13 and WT littermates at different time points during four weeks. The age of the mice is presented on the x-axis in days. **(B)** Weight of four-week-old Mov13 and WT littermates divided in males and females during four weeks. Values of standard deviations (SD) are shown for each group: WT (n=10, 50%male) and Mov13 (n=10, 50%male). Statistical significance is indicated on the graphs (P-values ≤0.05 (*), ≤ 0.01 (**)).

The initial average mean weight of the Mov13 mice and of their WT littermates at four weeks of age were 16.32 ± 1.28g and 17.69 ± 1.41g respectively. The final mean weight at eight weeks before sacrifice of the Mov13 mice was 20.27 ± 2.36g and of the wild-type littermates 21.8 ± 3.02g. Weights were not significantly different between Mov13 and WT mice (**Figure 1A**). However, we found a considerably lower weight in the male Mov13 mice compared to male WT mice (*p*<0.001) from the second week onwards (**Figure 1B**). In addition, mice at the age of 15 weeks started presenting symptoms of lymphoma (fluffy coat, jerky motor skills, reduced appetite, increased size of liver and spleen) which was confirmed by the analysis of the liver and spleen by pathology examination.

### 3.2 Increased cortical microCT measurements in Mov13 mice

**Figure 2:**
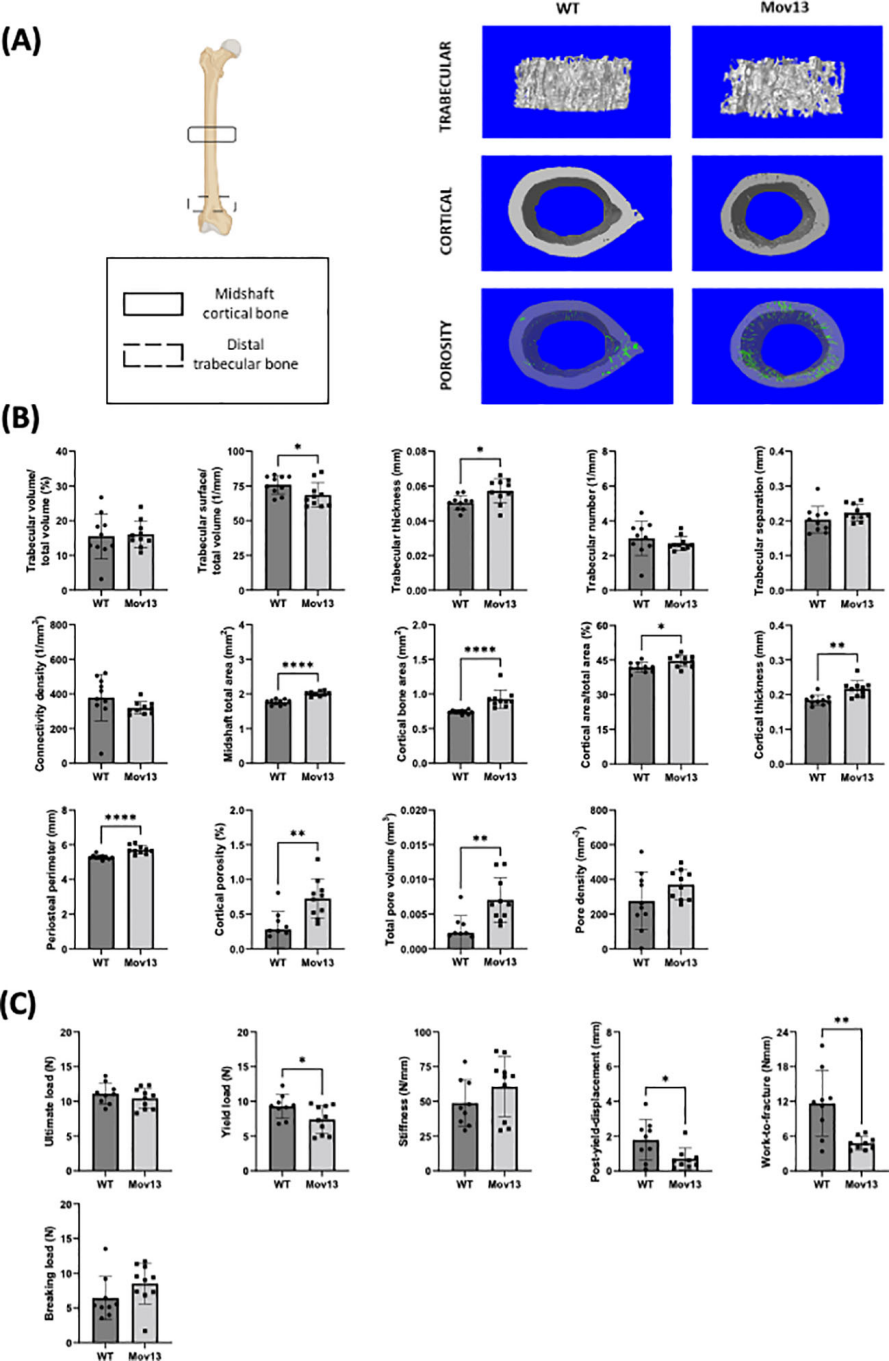
Biomechanical and structural measurements of the Mov13 mouse. **(A)** Schematic overview of femur showing the regions used to measure the midshaft cortical bone and distal trabecular bone and the 3D reconstructions of the microCT scans of the trabecular bone, cortical bone and cortical porosity of a wild-type and Mov13 mouse. **(B)** Trabecular and cortical parameters were measured from microCT scans, corrected for body weight, of Mov13 mice and wild-type littermates. Values of standard deviation (SD) are shown for each group: WT (n=10, 50%male), Mov13 (n=10, 50%male). **(C)** The biomechanical properties (Ultimate load, yield load, breaking load, stiffness, post-yield displacement and work-to-fracture) were measured using the load-displacement curve after 3-point bending and corrected for midshaft total area (Tt.Ar). Values of standard deviation (SD) are shown for each group: WT (n=9, 45%male), Mov13 (n=10, 50%male). Statistical significance is indicated on the graphs (P-values ≤0.05 (*), ≤ 0.01 (**), ≤0.0001 (****)). Tb.N, Ct.Ar and the breaking load were measured with a non-parametric unpaired T-test.

MicroCT scanning of the distal trabecular femoral bone and midshaft cortical femoral bone indicated significant differences in many structural parameters between Mov13 and WT littermates (**Figure 2A**). Significantly lower trabecular bone surface density (Tb. BS/TV) (*p*<0.05) and a significantly higher trabecular thickness (Tb.Th) (*p*<0.05) were detected in Mov13 mice (**Figure 2B**). Furthermore, Mov13 mice revealed a pattern of less trabecular number (Tb.N) and trabecular connectivity density (Conn.D) (**Figure 2B**). In addition, a trend of higher trabecular separation (Tb.Sp) was documented (**Figure 2B**). No difference was observed in trabecular bone volume fraction (Tb. BV/TV) (**Figure 2B**). In addition, Mov13 mice presented a significantly higher cortical midshaft total area (Tt.Ar) (*p*<0.001), cortical bone area (Ct.Ar) (*p*<0.001), cortical area fraction (Ct.Ar/Tt.Ar) (*p*<0.05), cortical thickness (Ct.Th) (*p*<0.01), periosteal perimeter (Ps.Pm) (*p*<0.0001), cortical porosity (Ct.Po) (*p*<0.01) and total pore volume (Po.V) (*p*<0.01) (**Figure 2B**). Moreover, a trend of a higher cortical pore density (Po.Dn) was also noted in Mov13 mice (**Figure 2B**). This data indicates a reduction in trabecular bone in contrast to increased formation of cortical bone with higher porosity in Mov13 mice.

### 3.3 Reduced bone strength in Mov13 femur

The biomechanical properties of the femur were measured by 3-point bending and calculated from the resulting load-displacement curve and corrected for the cortical midshaft total area (Tt.Ar). Mov13 mice presented a significantly lower yield load (*p*<0.05), post-yield displacement (*p*<0.05) and Work-to-fracture (*p*<0.001) compared to their WT littermates (**Figure 2C**). In addition, no significant difference in the ultimate load, stiffness and breaking load was detected in Mov13 mice a trend of higher stiffness and breaking load (**Figure 2C**). A reduced PYD and WTF in Mov13 mice was expected considering the lower yield load. This suggests that Mov13 femora are more brittle due to a reduced amount of load needed before the bone suffers permanent damage and reduced ductility measured by PYD.

### 3.4 Histomorphometric parameters of Mov13 tibia

None of the histomorphometric parameters presented significant differences between Mov13 and WT mice (**Figure 4**). However, there was a trend of a lower cortical width (Ct.Wi), higher trabecular width (Tb.Wi) and higher osteoid perimeter (O.Pm/B.Pm) in Mov13 compared to WT mice (**Figure 4B**).

**Figure 3:**
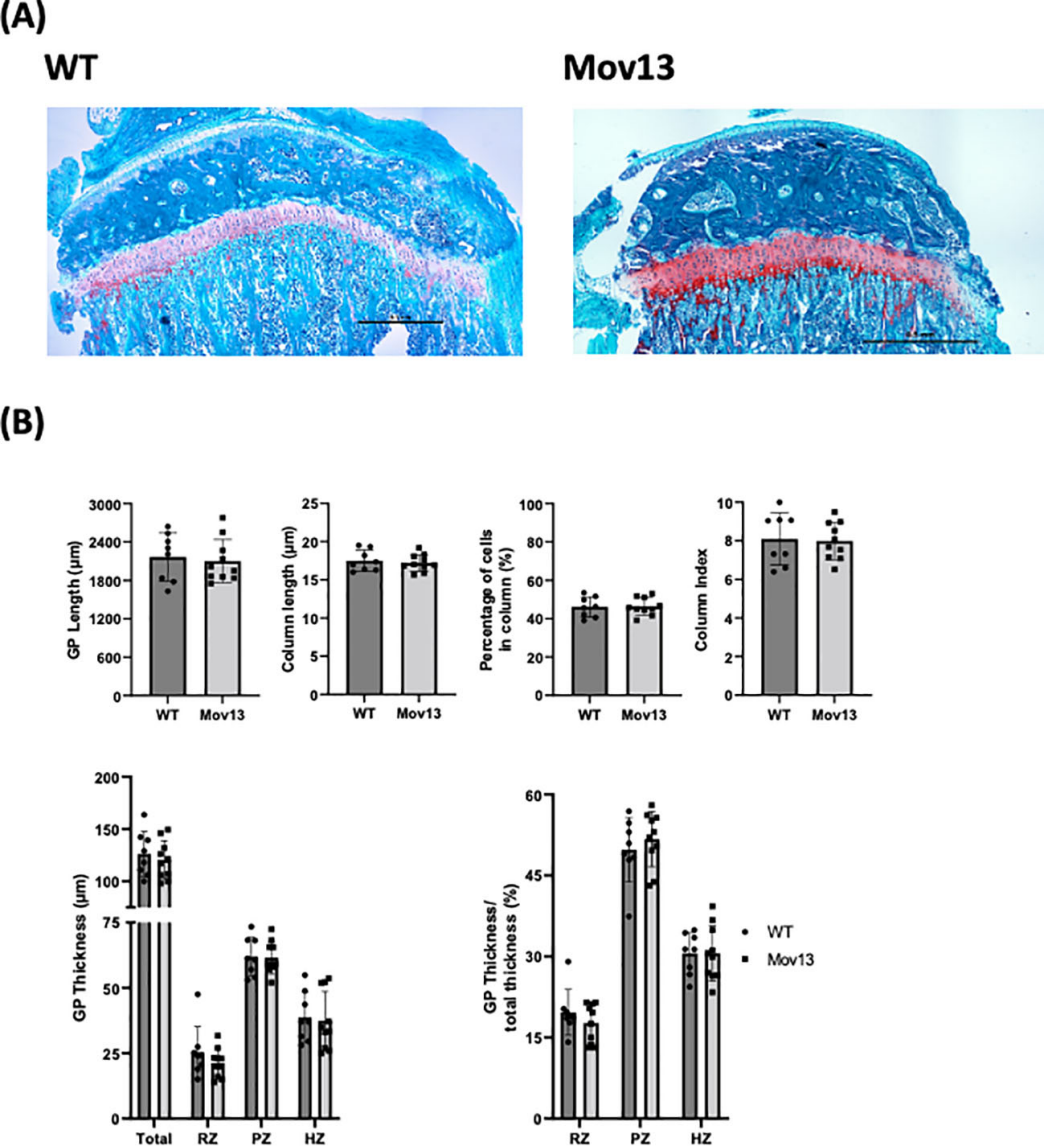
Histomorphometrical analysis of mouse growth plate. **(A)** Representative images of Safranin O staining of left tibia from eight-week-old Mov13 mice and WT littermates (10x10 magnification). **(B)** GP length, column length, column index (CI), percentage of cells in column, thickness (total and the different zones; resting zone (RZ), proliferative zone (PZ) and hypertrophic zone (HZ)) and ratio of thickness. Values of standard deviation (SD) are shown for each group: WT (n=8, 40%male) and Mov13 (n=10, 50%male). Statistical significance is indicated on the graphs (P-values ≤0.05 (*)). GP thickness RZ and PZ were measured with a non-parametric unpaired T-test.

**Figure 4:**
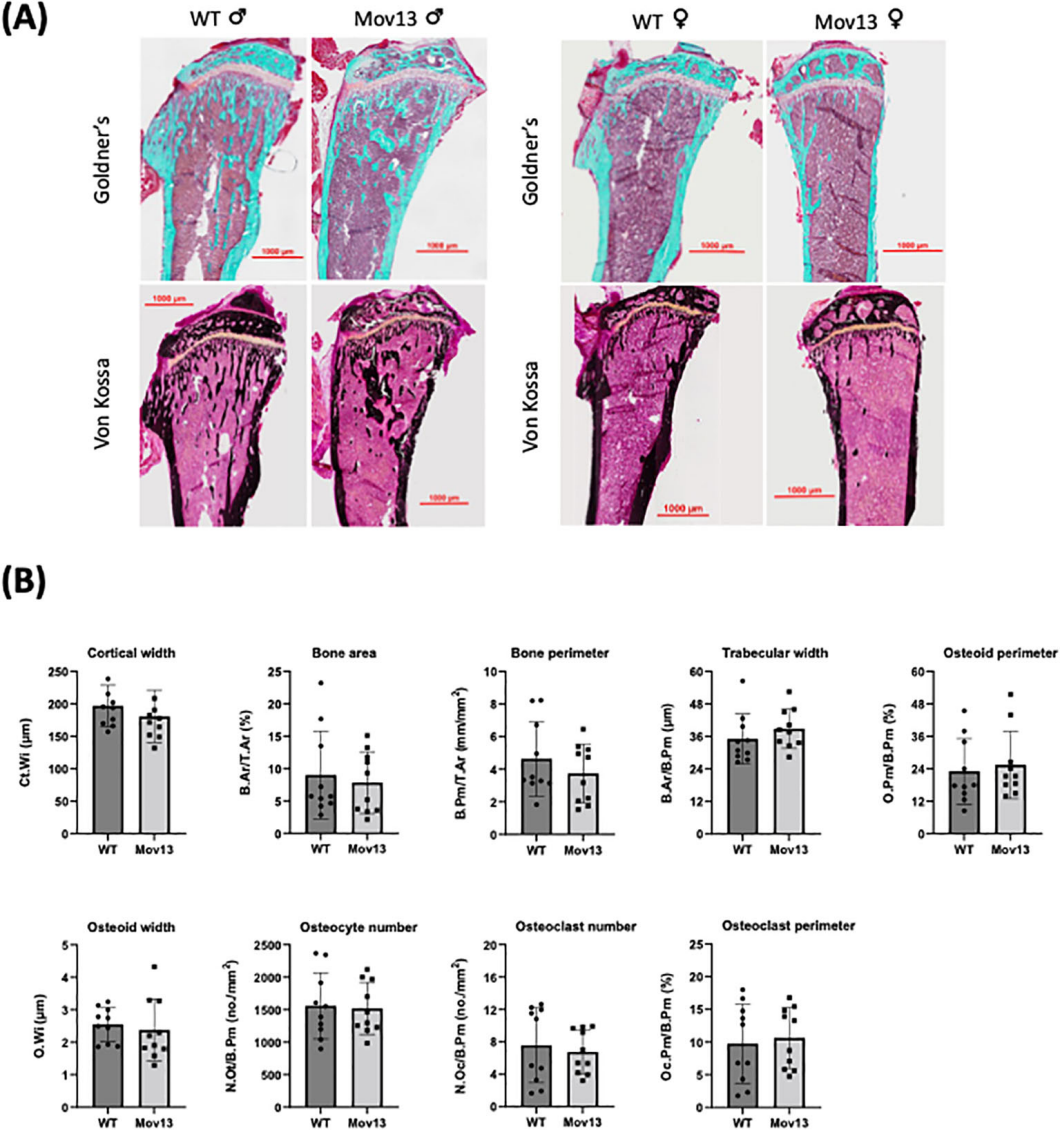
Histomorphometrical analysis of Mov13 and WT tibias. **(A)** Representative images of Mov13 and WT mice tibia bone histology. Masson Goldner’s trichrome (10x objective) and Von Kossa (20x objective) staining of male and female mice tibiae. **(B)** Cortical and trabecular measurements of Mov13 and WT tibias: Cortical width (Ct.Wi), Bone area (B.Ar/T.Ar), Bone perimeter (B.Pm/T.Ar), Trabecular width (B.Ar/B.Pm), Osteoid perimeter (O.Pm/B.Pm), Osteoid width (O.Wi), Number of osteocytes (N.Ot/B.Pm), Number of osteoclasts (N.Oc/B.Pm), Osteoclast perimeter (Oc.Pm/B.Pm). Values of standard deviation (SD) are shown for each group: WT (n=10, 50%male), Mov13 (n=10, 50%male). Statistical significance is indicated on the graphs (P-values ≤0.05 (*)). Bv/Tv, B.Pr/T.Ar and O.Wi were measured with a non-parametric unpaired T-test.

We analyzed the growth plate (GP), since morphological GP changes were previously found in OI patients (29). However, our GP analysis revealed no significant differences between the Mov13 and WT mice for any of the measurements (**Figure 3**). However, a discernible lower total growth plate thickness was detected in Mov13 mice (**Figure 3B**). We also perceived a slightly lower ratio for the resting zone of Mov13 mice, no difference was discovered for the hypertrophic zone. Though, we found a marginally higher ratio of the proliferative zone in the Mov13 mice compared to the WT mice (**Figure 3B**).

### 3.5 Bone formation marker is reduced in Mov13 mice

**Figure 5:**
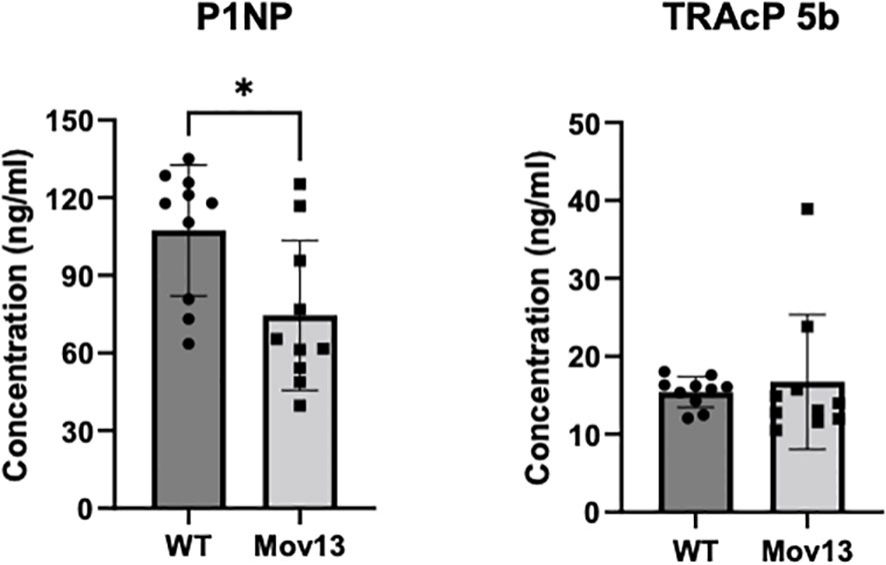
ELISA of bone turnover markers in serum. Markers for bone formation (P1NP) and bone resorption (TRAcP 5b) were measured in mice blood serum with ELISA. P1NP and TRAcP 5b measurement of eight-week-old Mov13 and WT littermates. Standard deviations (SD) are shown for each group: WT (n=10, 50%male) and Mov13 (n=10, 50%male). Statistical significance is indicated on the graphs (P-values ≤0.05 (*)). TRAcP 5b was measured with a non-parametric unpaired T-test.

Serum P1NP levels, a bone formation marker, were 36.13% lower in the 8-week-old Mov13 than in WT mice, 74.55 ± 28.93ng/ml and 107.43 ± 25.33ng/ml respectively (*p*<0.05) (**Figure 5**). We did not observe a difference in the bone resorption marker, TRAcP 5b, between Mov13 and WT mice (**Figure 5**). This substantiates the expected reduced Col1a1 production in Mov13 mice and might indicate an overall reduced bone formation.

### 3.6 Expression of *Col1a1*, osteogenic, collagen and immune marker genes in bone tissue of Mov13 mice

**Figure 6:**
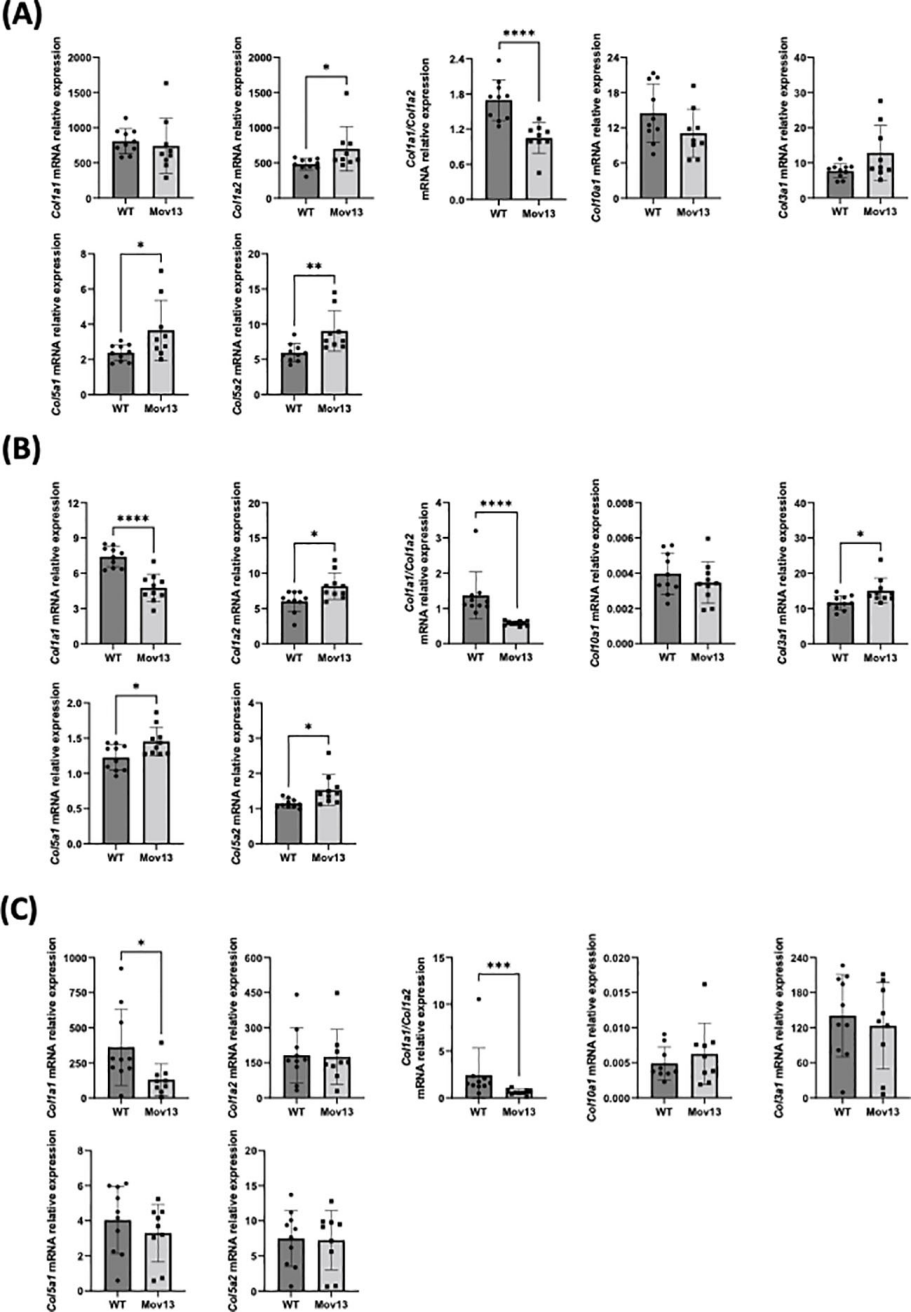
Relative mRNA expression of collagens in bone, heart and skin tissues. Relative mRNA expression of different collagen types in **(A)** bone, **(B)** heart and **(C)** skin tissue of Mov13 and WT mice. Relative mRNA expression is represented as ΔΔCt values. P-values are indicated as follows: (P-values ≤0.05 (*), ≤ 0.01 (**), ≤ 0.001 (***), ≤0.0001 (****)). Bone *Col1a2*, *Col5a2*, *Col1a1/Col1a2*, heart *Col1a2*, *Col3a1*, *Col1a1/Col1a2*, skin *Col1a1*, *Col10a1 and Col1a1/Col1a2* were measured with a non-parametric unpaired T-test.

No difference in transcriptional expression of *Col1a1* was measured between Mov13 and WT mice. However, the *Col1a1/Col1a2* ratio was significantly lower (*p*<0.001) in the overall Mov13 group, due to higher *Col1a2* expression in Mov13 (*p*<0.05) (**Figure 6A**). Surprisingly, Mov13 mice also showed upregulation of collagen type V: *Col5a1*, *Col5a2* (*p*<0.05 and *p*<0.01 respectively); osteogenic and related markers: *Bglap* (*p*<0.05), *Edn1* (*p*<0.01), *Eln* (*p*<0.05), *Fgf23* (*p*<0.001) and immune marker *Smad7* (*p*<0.01) (**Figure 6A**, **Supplemental Figures 3, 5A**). Several common differentially expressed genes in osteogenesis imperfecta mouse (oim) and Col1a1^Jrt/+^ mice were also tested (30). None of these genes (*Cgref*, *Cyp2e1*, *Ifitm5*, *Rerg*, *Slc13a5*, *Smpd3*, *Gypa*) presented expression differences in Mov13 compared to WT, highlighting differences in bone fragility mechanisms of DN and HI OI mouse models (**Supplemental Figures 3, 5A**). In addition, many other osteogenic and related markers (*Fn1*, *Opg*, *Rankl*, *Runx2*, *Sost*, *Spp1*, *Tgfβ*, *Agtr1a*, *Fbn1)* and immune markers (*Il10*, *Il1b*, *Il6*, *Serpine1*, *Tnfα*) did not present any difference between Mov13 and WT mice (**Supplemental Figure 3**).

### 3.7 Expression of *Col1a1* and target genes in heart and skin tissue of Mov13 mice

Heart tissue presented a 35.76% lower expression of *Col1a1* (*p*<0.0001) and a lower *Col1a1/Col1a2* ratio (*p*<0.01) in Mov13 mice. Mov13 mice appeared to have a significantly higher expression of other collagens *(Col1a2*, *Col3a1*, *Col5a1*, *Col5a2)* (*p*<0.05), cardiological markers (*Tbx20* (*p*<0.05), *Spp1* (*p*<0.05)) and immune markers (*Tnfα* (*p*<0.05), *Il10* (*p*<0.05)). As in the bone tissue, several common differentially expressed genes (*Cgref*, *Cyp2e1*, *Ifitm5*, *Slc13a5*, *Smpd3*, *Gypa*) of the oim and Col1a1^Jrt/+^ mice were measured in the heart and presented no difference between Mov13 and WT mice (**Supplemental Figures 4, 5B**). However, the *Rerg* gene expression was significantly lower in Mov13 mice compared to WT (*p*<0.05) (**Figure 6B**, **Supplemental Figures 4, 5B**). No difference was seen in the other cardiological and related markers (*Agtr1a*, *Tnnt2*, *Gata4*, *Edn1*, *Eln*), immune markers (*Il1b*, *Il6*, *Smad7*, *Serpine1*) and osteogenic markers (*Fn1*, *Tgfβ*) (**Supplemental Figures 4, 5B**). Similarly to heart tissue, *Col1a1* in the skin of Mov13 mice was reduced by 63.58% (*p*<0.05), (**Figure 6C**). No difference was observed in the expression of other collagen genes in the skin (**Figure 6C**, **Supplemental Figure 5C**).

## 4 Discussion

In this study, a thorough characterization of heterozygous Mov13 mice with a null mutation in the alpha1 collagen type I locus is provided. The reduced mechanical and material properties in the Mov13 mice compared to healthy litter-mate controls were demonstrated with microCT and 3-point bending. No significantly lower expression of *Col1a1* mRNA level was detected in Mov13 mineralized connective tissue, although this was the case in the heart and skin tissue.

The most important measurement in support of Mov13 as a suitable mouse model for HI OI type 1, is the expected reduced mechanical properties of its long bones. Based on previous loading test outcomes on Mov13 femora, a distinctly lower work-to-fracture in Mov13 were expected (10, 11, 15, 31). It has to be stated that all previous loading tests in Mov13 mice were performed with 4-point bending; as such, our findings with 3-point bending are not entirely directly comparable. However, in addition to a significantly lower work-to-fracture, post-yield displacement and yield load, a trend of higher breaking load and stiffness in Mov13 mice were also detected, corroborating previous studies about the causative role of reduced collagen type I in bone fragility (10, 15, 31). All together, we can hypothesize that the bone brittleness is caused by a reduced tissue bending strength and ductility resulting in a a lower yield load, post-yield displacement and work-to-fracture (15).

A lower trabecular bone surface density (Tb. BS/TV) and significantly higher trabecular width, were measured in Mov13 mice with microCT. The difference in trabecular BS/TV could suggest a possible alteration in the tendency of the trabecular tissue for microstructure remodeling, caused by a reduced available surface for osteoclasts and osteoblasts for replacing old and damaged bone matrix (32). It can be hypothesized that this reflects a mechanism by which bone tissue may compensate for this with an increase in trabecular thickness. Even though no other significant differences were found in the trabecular bone, we did observe a different trabecular bone structure with few but large trabeculae based on a lower trabeculae number, connectivity density and a higher trabecular separation and the previously mentioned lower Tb. BS/TV and higher Tb.Th. However, a difference in the trabecular bone volume fraction (Tb. BV/TV) was not observed. In agreement with previous studies, both the cortical bone area and cortical porosity were higher in the Mov13 mice (11, 15, 31). In addition, higher midshaft total area, cortical area fraction, cortical thickness, total pore volume and periosteal perimeter in Mov13 mice were noted. Hence, the hypothesis of Bonadio et al. and Jepsen et al, that the bone tissue is adapting *via* the periosteal surface to compensate for the loss of load-bearing capacity due to the altered microstructure associated with a higher porosity in Mov13 mice was substantiated by our data (11, 15, 31).

No significant differences were found in both cortical and trabecular histomorphometrical measurements. However, this did not correlate to our significantly higher cortical measurements determined by microCT. Yet, a trend of decreased trabecular bone was determined by both histomorphometry and microCT. This was in contrast to the reduced mechanical properties noted in the Col1a1 ^± 365^ mouse model, in which the underlying cause was attributed to a lower and sparser amount of trabecular bone and a higher number of osteoclasts instead of changes in the cortical bone (6). In addition, no difference in any of the osteoid parameters was detected; this is in contrast with findings in OI patients, including HI OI type 1, in which a difference in osteoid surface has been reported (33, 34). This clearly emphasizes the importance of measuring the bone phenotype not only histomorphometrically but also based on structural parameters by microCT. In addition, this might also demonstrate that more tissue sections are necessary to analyze in the future. Sanguinetti et al. found a disruption in the growth plate in OI type 1 and 3 patients due to an increased thickness of the hypertrophic zone and shorter columns in the proliferative zone (26, 29). However, we failed to find any significant differences in any growth plate measurement between Mov13 and WT mice.

Our results did not reveal any difference in weight between Mov13 and WT mice. However, significantly lower weight was observed in Mov13 males in agreement with male Col1a1 ^± 365^ mice (6). This warrants further research to further explain the lack of weight difference between female WT and Mov13 mice.

Significantly lower P1NP concentration was noted in Mov13 mice. Since almost 90% of the organic portion of the bone matrix consists of type I collagen and P1NP is cleaved off when collagen type I is secreted, P1NP can be perceived as a good marker for collagen type I production and bone formation (35). As such, a decreased bone formation was clearly indicated by the lower P1NP concentration in Mov13 mice which corresponds with the lower concentration found in HI OI type 1 patients and Col1a1 ^± 365^ mice (6, 36). No difference in TRAcP 5b concentrations was found in Mov13 mice. TRAcP 5b measurement informs about osteoclast number rather than osteoclasts activity and as such corroborates our histomorphological findings in the tibia (37, 38). In comparison, an increased number of TRAP-positive osteoclasts was observed in the trabecular femur of eight-week-old Col1a1 ^± 365^ mice (6). In addition, equal levels in the gene expression of osteoclast related markers, *Opg* and *Rankl*, were also noted between Mov13 and WT mice. Conclusively, the lower level of P1NP is in line with the expected reduction in collagen type I synthesis in Mov13 mice but more investigation is needed to fully comprehend the catabolic mechanism involved in the bone turnover of HI OI.

Despite transcription inactivation of one *Col1a1* allele by MMuLV, we did not detect a lower *Col1a1* mRNA expression by qPCR in the bone tissue of heterozygous Mov13 mice. In agreement with our findings a previous study reported a 30% increase in *Col1a1* gene expression by northern blotting in the femur of eight-week-old Mov13 mice and returned to a comparable level at 15 weeks old (31). This is in contrast with Col1a1 ^± 365^ mice where a decreased pro-α1(I) expression in femur of eight-week-old mice was measured (6). Additionally, in the tibia of 17-week-old Mov13 mice a nine-fold *Col1a1* reduction was observed by qPCR (18). Similarly, lower expression of *Col1a1* was observed in otic capsule, while parietal bone *Col1a1* expression was almost 2-fold higher compared to WT littermates (18). Though, Mov13 embryos and embryonic fibroblast cultures also did not reveal a convincing decrease in *Col1a1* expression by northern blotting (39). In comparison with Iruela-Arispe et al., a significantly lower *Col1a1* expression was measured in total skin of our Mov13 mice. Regardless of mRNA expression findings, Mov13 skin fibroblasts from 15-week-old mice have been shown to produce half the normal amount of radiolabeled type I collagen (6, 10). In addition, decreased collagen is recorded in the supernatant and extracellular matrix of the embryonic Mov13 fibroblast cultures (39). Finally, although no reduced *Col1a1* expression was measured in the bone tissue of Mov13 mice, as mentioned above, a decreased P1NP concentration was detected. As such, this indicates a divergence between *Col1a1* gene expression and the eventual collagen type I secretion in the bone tissue. Furthermore, the high variability in *Col1a1* gene expression in the different studies and different bone tissues (femora, tibiae and otic capsule) also hamper the validation of this mouse model for HI OI type 1.

Regarding the expression of other collagen types in fibroblast-like cells of homozygous Mov13 mice, no compensation was seen on protein level (40). Unfortunately, our study could not include protein measurement of collagen types and the correlation between *Col1a1* mRNA and protein expression has previously only been examined in Mov13 fibroblasts (39). Consequently, it remains unclear what the *Col1a1* mRNA expression changes mean on protein level in the different Mov13 tissues. Equally lacking is the biological significance of the increased expression of *Col1a2*, *Col5a1*, *Col5a2* and *Col3a1* in the bone and heart tissues of Mov13 mice. We can hypothesize that the reduction of Col1a1 could influence the expression of genes encoding the fibril-forming collagens type III and V with which it forms fibrils in the bone and myocardium (41, 42). However, this has not been shown before as no difference in *Col1a1* expression levels were found in a Ehlers-Danlos syndrome HI Col5a1^+/-^ mouse model (43).

No change was found in *Runx2* expression whereas *Bglap* expression was higher in Mov13 mice. In contrast, lower expression of *Runx2* and *Bglap* were found in the femur of eight-week-old Col1a1 ^± 365^ mice. However collagen type I regulates osteoblast differentiation, which may potentially justify this downregulation in the Col1a1 ^± 365^ mice (6, 44). Notably, a higher expression of *Smad7*, which is an inhibitor of TGFβ signaling, was also found in the Mov13 mice (45). Excessive TGFβ signaling and bioactivity have previously been found in OI mouse models (*Crtap* ^-/-^ and *Col1a2* ^tm1.1Mcbr^) and serum of OI patients respectively (46, 47). It can be speculated that *Smad7* upregulation may oppose potential TGFβ activity. Recent studies have hinted towards the presence of inflammation in OI patients (48, 49). No upregulation of inflammation factors was identified in bone tissue, whereas *TNFα*, *Il10*, *Tbx20* and *Spp1*, were upregulated in heart tissue. All four genes play a crucial role in cardiac remodeling, revealing signs of possible cardiac dysfunction caused by significant reduction of *Col1a1* expression in the hearts of Mov13 mice (50–53). Dysregulated expression of estrogen signaling regulator *Rerg* was previously reported in bone of oim and Col1a1^Jrt/+^ mice. However, in contrast to mice with DN defect, Mov13 have significantly downregulated *Rerg* expression in heart.

A concerning factor interfering with gene expression may be the presentation of severe lymphoma in adult Mov13 mice. Even though the mice only visibly started deteriorating starting from 15 weeks old on, it cannot be excluded that the gene expression was affected by a precancerous state. Notably, in addition to a high variability in bone tissue, which was shown in the literature and our results, a significant decrease in *Col1a1* gene expression was detected in the heart and skin tissue of our Mov13 mice. Even though the heart and skin may be less likely affected by lymphoma, given the severe nature of the disease, it is not possible to safely conclude if these gene expression changes can be attributed to cancer or tissue-dependent collagen regulation as reported in Mov13 odontoblasts (18, 54, 55). The *Col1a1* expression variability present in Mov13 mice can also be explained with the presence of cell-specific, orientation-dependent transcriptional elements in the first intron of the *Col1a1*, known to exist in both mice and human genomes (4, 56–60). It is also worth to note that no obvious evidence of lymphoma was detected by the analysis of our mice, given that there was no severe trabecular and cortical bone loss, no decreased osteoclast number, serum TRAcP 5b concentration or no downregulation of *Runx2* and *Bglap* detected (61, 62). However, elevated levels of *Fgf23* expression, found in our Mov13 mice and which is known to suppress mineralization of osteoblasts and negatively regulate bone homeostasis, are associated with acute myeloid leukemia (63–66). The higher expression of *Edn1* and *Eln* could also underline metastatic processes in the bone (67–69). On the other hand, it could be plausible that the upregulation of both genes is a consequence of the bone defect in Mov13 mice. Similarly to Mov13, oim mice have shown upregulation of *Edn1*, important for angiogenesis, osteoblastic proliferation and bone development, whereas *Eln* overexpression might be an attempt to compensate for the loss of bone extracellular matrix elasticity *(* 30 *).* High content of elastin was shown in biopsies of human OI type 4 and Ehlers-Danlos syndrome patients *(* 70, 71 *).* Due to the mixed effect of the transgenesis and possible development of lymphoma, the biological interpretation of the gene expression pattern in Mov13 mice remains unclear.

In conclusion, our findings corroborate that the Mov13 mice present a reduced mechanical strength and altered material properties in accordance to the expected reduced Col1a1 in the bone tissue. Unfortunately, the presentation of severe lymphoma in adult mice and the challenging variability of the Mov13 bone phenotype prevents us from recommending this mouse model for therapeutic studies for of HI OI type 1, especially when older mice are required. A new reliable mouse model, which recapitulates HI OI type 1, is clearly urgently needed to facilitate mild OI research and the testing of new therapies.

## Data availability statement

The original contributions presented in the study are included in the article/**Supplementary Material**. Further inquiries can be directed to the corresponding author.

## Ethics statement

The animal study was reviewed and approved by the Central Committee of Animal experiments (CCD) of the of the Netherlands and in agreement with the Animal Welfare Body of the Amsterdam UMC in full compliance with the directive 2010/63/EU.

## Author contributions

All authors have contributed substantially to the conception of the article and critical revision. LC, together with the help of HE, performed all the experimental work for **Figures 1**, **2** and **supplemental Figures 1 and 2**. LZ and LC together performed all the experiments for **Figures 3**, **4**. LZ, together with the help of LW, performed all the experimental work and analysis for **Figure 6** and **supplemental Figures 3–5**. LC performed the analysis for **Figures 1**–**3**, **5** while LZ also performed the analysis for **Figure 4**. DM, NB, GP and EE supervised the work of LC, LZ, LW and HE and contributed to the experimental design and writing of the manuscript. All authors contributed to the article and approved the submitted version.

## Glossary

**Table:**

ASBMR	American society for bone and mineral research
B.Ar	Bone area
B.Ar/T.Ar	Bone area
B.Pm	Bone perimeter
B.Pm/T.Ar	Bone perimeter
BTM	Bone turnover markers
CCD	Central Committee of Animal experiments
Conn.D.	Connectivity density
Ct.Ar	Cortical bone area
Ct.Ar/Tt.Ar	Cortical area fraction
Ct.Po	Cortical porosity
Ct.Th	Average cortical thickness
Ct.Wi	Cortical width
Ct.Wi	Cortical width
d	Displacement
DEC	Animal experiment committee
DN	Dominant negative
F	Force
GP	Growth plate
HI	Haploinsufficiency
HZ	Hypertrophic zone
microCT	Micro computed tomography
MMA	Methylmethacrylate
MMuLV	Moloney murine leukemia virus
N.Oc	Osteoclast number
N.Oc/B.Pm	Number of osteoclasts
N.Ot	Osteocyte number
N.Ot/B.Pm	Number of osteocytes
O.Pm	Osteoid perimeter
O.Pm/B.Pm	Osteoid perimeter
O.Wi	Osteoid width
Oc.Pm	Osteoclast perimeter
OI	Osteogenesis Imperfecta
P1NP	Procollagen 1 Intact N-Terminal Propeptide
Po.Dn	Pore density
Po.V	Total pore volume
PYD	Post-yield displacement
PZ	Proliferative zone
RZ	Resting zone
T.Ar	Tissue area
Tb. BS/TV	Trabecular bone surface density
Tb. BV/TV	Trabecular bone volume fraction
Tb.N	Trabecular number
Tb.S	Trabecular separation
Tb.Th	Trabecular thickness
Tb.Wi	Trabecular width
TracP 5b	Tartrate-resistant acid phosphatase 5b
TRAP	Tartrate-resistant acid phosphatase
Tt.Ar	Total cross-sectional area inside the periosteal envelope
WT	Wild-type
WTF	Work-to-fracture
