# Supplementary material for: Exploration of the skeletal phenotype of the Col1a1 +/Mov13 mouse model for haploinsufficient osteogenesis imperfecta type 1
Source: Front Endocrinol (Lausanne). 2023 Mar 8;14:1145125. doi: 10.3389/fendo.2023.1145125 (PMC10031054; doi:10.3389/fendo.2023.1145125)
Supplement: Supplementary file 1 [file DataSheet_1.docx]

Supplementary Material

**Exploration of the skeletal phenotype of the *Col1a1*^+/Mov13^ mouse model for haploinsufficient Osteogenesis Imperfecta type 1**

**Lauria Claeys^1^, Lidiia Zhytnik^1,2^, Lisanne E. Wisse^1^, Huib W. van Essen^3^, E. Marelise W. Eekhoff^4^, Gerard Pals^1^, Nathalie Bravenboer^3†^, Dimitra Micha^1†*^**

† These authors contributed equally to this work and share last authorship

^1 Department of Human Genetics, Amsterdam Movement Sciences, Tissue function & Regeneration and Musculoskeletal health, Amsterdam UMC location Vrije Universiteit Amsterdam, Amsterdam, The Netherlands^

^2 Department of Traumatology and Orthopeadics, Institute of Clinical Medicine, The University of Tartu, Tartu, Estonia^

^3 Department of Clinical Chemistry, Amsterdam Movement Sciences, Tissue function & Regeneration and Ageing & Vitality, Amsterdam UMC location Vrije Universiteit Amsterdam, Amsterdam, The Netherlands^

^4 Department of Endocrinology and Metabolism, Amsterdam Rare Bone Disease Center, Amsterdam UMC, Amsterdam, The Netherlands^

^Dimitra Micha*^

[^d.micha@amsterdamumc.nl^](mailto:d.micha@amsterdamumc.nl)

**Supplemental Figure 1**

Measurements of growth plate on Safranin-O stained mice tibia. Length of the growth plate (light green line), resting zone (dark green line), proliferative zone (dark blue line), hypertrophic zone (light blue line), length of the column (yellow line), cells inside column (green triangles), cells outside columns (yellow dots) and angle measurement (green/blue arrow).

**Supplemental Figure 2**

Typical load-displacement curve resulting from a loaded femur till breaking with 3-point bending and the calculated parameters from this curve: Ultimate load, Breaking load, Yield load, Stiffness, Post-yield displacement and Work-to-fracture.

**Supplemental Figure 3**

## Relative mRNA expression of osteogenic genes in humeri and femur of Mov13 and WT mice. Relative mRNA expression is represented as ΔΔCt values. Values of standard deviation (SD) are shown for each group: WT (n=8, 50%male) and Mov13 (n=9, 60%male). P-values ≤0.05 (*), ≤ 0.01 (**), ≤ 0.001 (***)). *Runx2, Serpine, Il10, Tgfβ* and *Cyp2e1* were measured with a non-parametric unpaired T-test.

**Supplemental Figure 4**

## Relative mRNA expression of cardiological and immune marker genes in heart ventricle of Mov13 and WT mice. Relative mRNA expression is represented as ΔΔCt values. Values of standard deviation (SD) are shown for each group: WT (n=10, 50%male) and Mov13 (n=10, 50%male). P-values ≤0.05 (*), ≤0.0001 (****). *Il10, Edn1* and *Cyp2e1* were measured with a non-parametric unpaired T-test.

**Supplemental Figure 5**

Relative mRNA expression in a heatmap of collagens genes, osteogenic genes, cardiological and immune marker genes in (A) humeri and femur (B) heart ventricles and (C) abdominal total skin of Mov13 and WT mice. Heatmaps display normalized ΔΔCt values in percentages.
