# Supplementary material for: Exploration of the skeletal phenotype of the Col1a1 +/Mov13 mouse model for haploinsufficient osteogenesis imperfecta type 1
Source: Front Endocrinol (Lausanne). 2023 Mar 8;14:1145125. doi: 10.3389/fendo.2023.1145125 (PMC10031054; doi:10.3389/fendo.2023.1145125)
Supplement: Supplementary file 2 [file Presentation_1.pptx]

## Slide 1
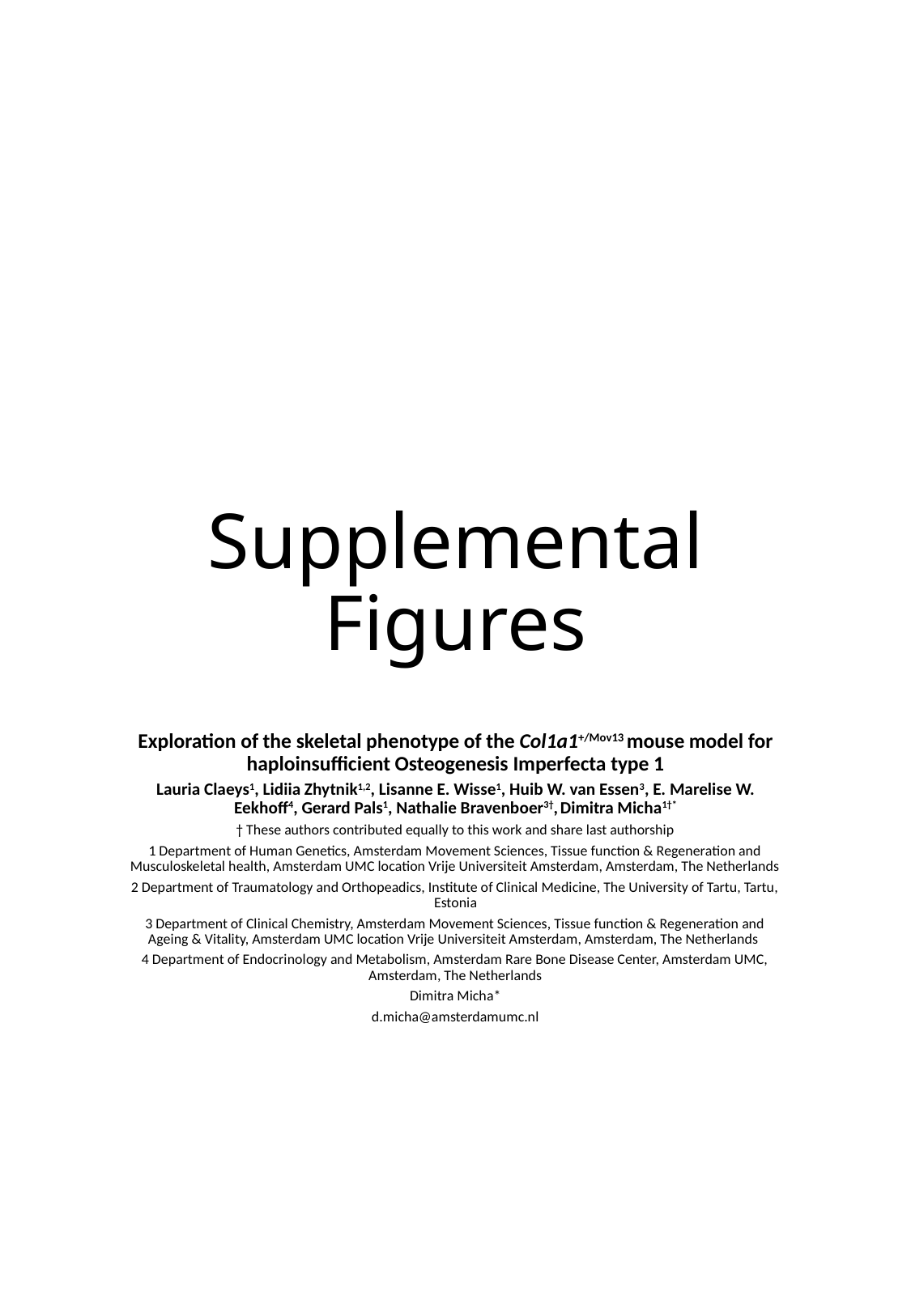

# Supplemental Figures
Exploration of the skeletal phenotype of the Col1a1+/Mov13 mouse model for haploinsufficient Osteogenesis Imperfecta type 1
Lauria Claeys1, Lidiia Zhytnik1,2, Lisanne E. Wisse1, Huib W. van Essen3, E. Marelise W. Eekhoff4, Gerard Pals1, Nathalie Bravenboer3†, Dimitra Micha1†*
† These authors contributed equally to this work and share last authorship
1 Department of Human Genetics, Amsterdam Movement Sciences, Tissue function & Regeneration and Musculoskeletal health, Amsterdam UMC location Vrije Universiteit Amsterdam, Amsterdam, The Netherlands
2 Department of Traumatology and Orthopeadics, Institute of Clinical Medicine, The University of Tartu, Tartu, Estonia
3 Department of Clinical Chemistry, Amsterdam Movement Sciences, Tissue function & Regeneration and Ageing & Vitality, Amsterdam UMC location Vrije Universiteit Amsterdam, Amsterdam, The Netherlands
4 Department of Endocrinology and Metabolism, Amsterdam Rare Bone Disease Center, Amsterdam UMC, Amsterdam, The Netherlands
Dimitra Micha*
d.micha@amsterdamumc.nl

## Slide 2
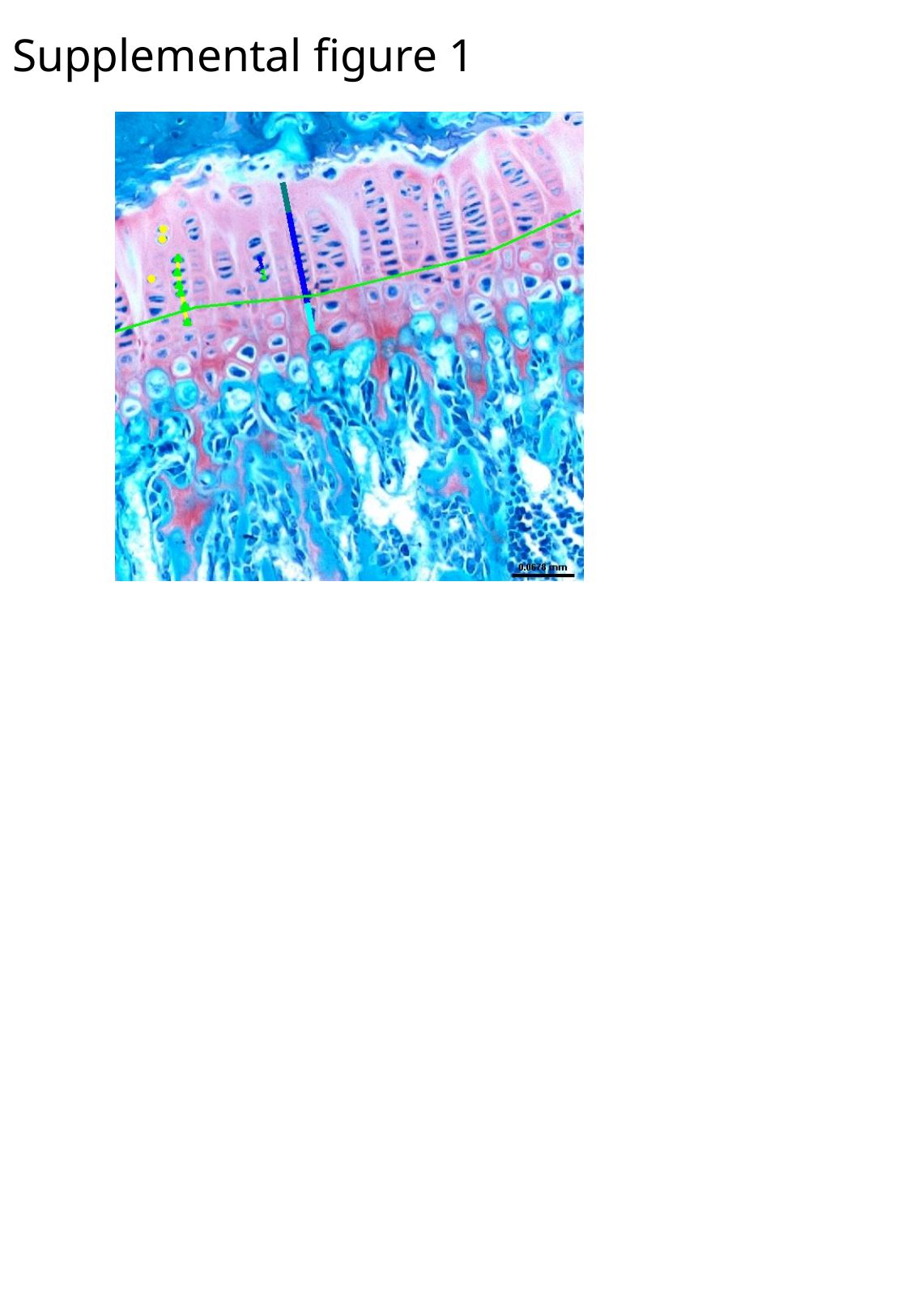

Supplemental figure 1

## Slide 3
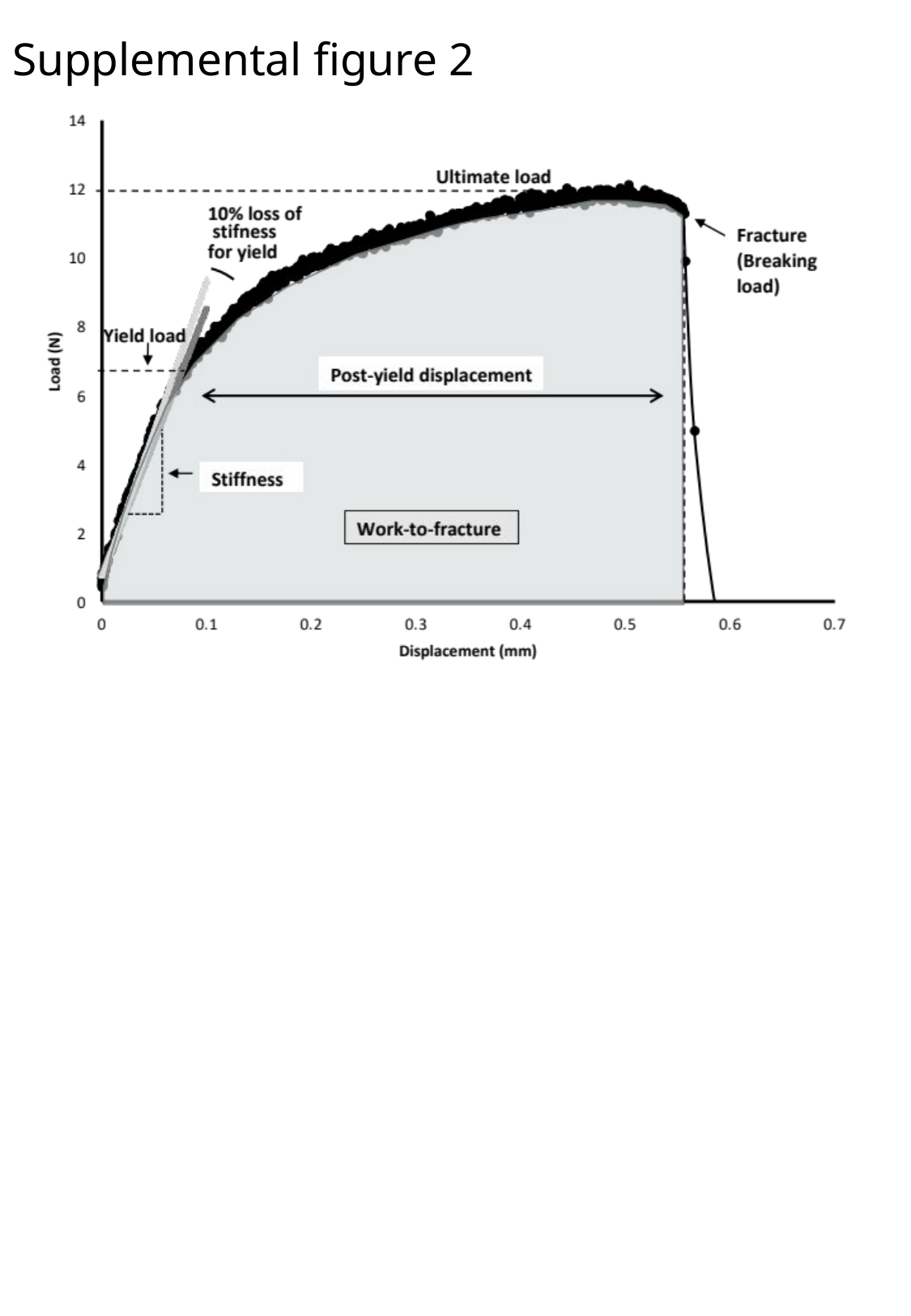

Supplemental figure 2

## Slide 4
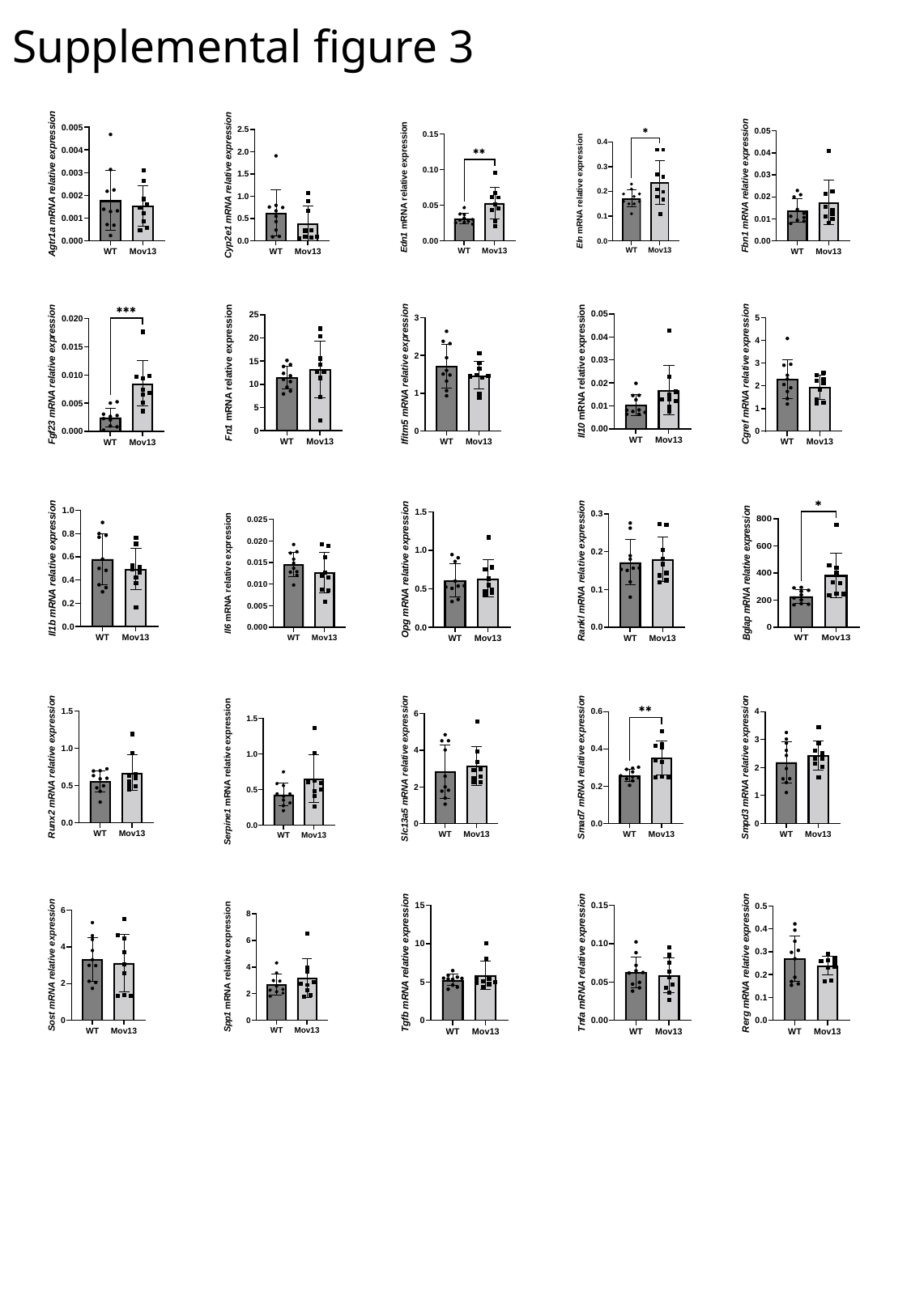

Supplemental figure 3

## Slide 5
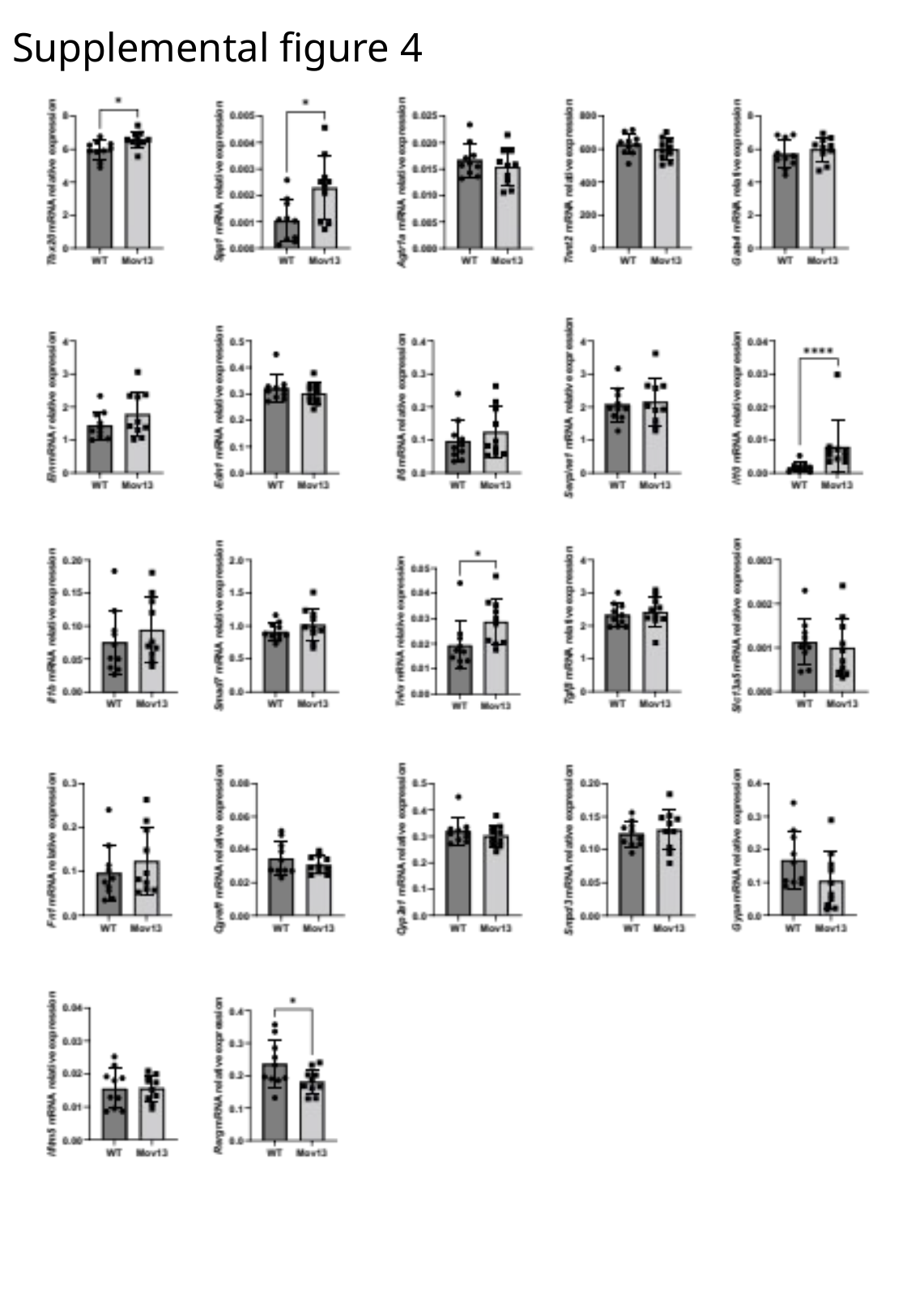

Supplemental figure 4

## Slide 6
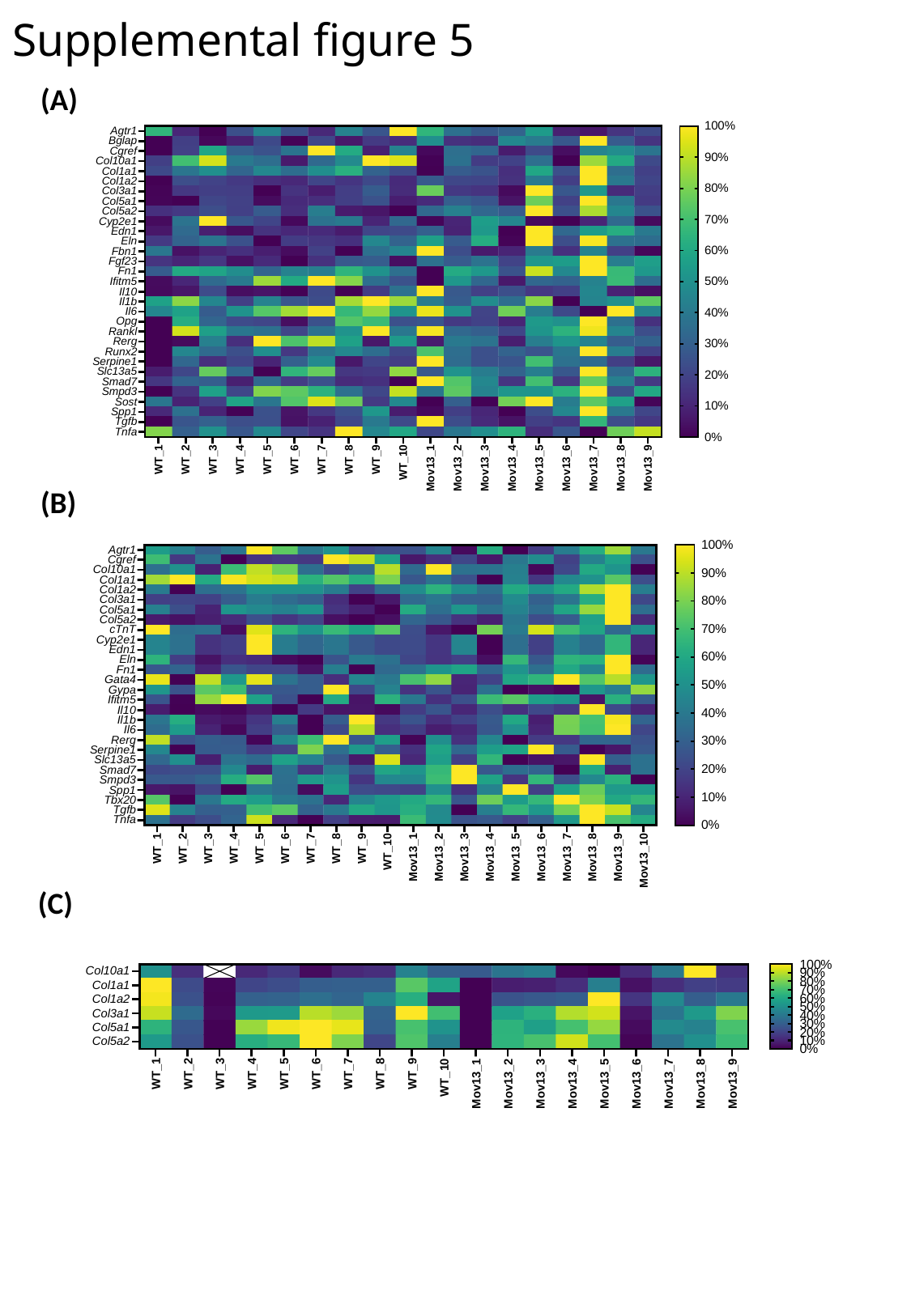

Supplemental figure 5
(A)
(B)
(C)
